# Supplementary material for: Design of AIEgens for near-infrared IIb imaging through structural modulation at molecular and morphological levels
Source: Nat Commun. 2020 Mar 9;11:1255. doi: 10.1038/s41467-020-15095-1 (PMC7062876; doi:10.1038/s41467-020-15095-1)
Supplement: Supplementary file 1 — Supplementary Information [file 41467_2020_15095_MOESM1_ESM.pdf]

Supplementary Information

Design of AIEgens for near-infrared IIb imaging through structural modulation at molecular and morphological levels

Li *et al*.

Materials. All the chemicals and reagents were purchased from chemical sources, and the solvents for chemical reactions were distilled before use. Benzo[1,2-c:4,5-c′]bis([1,2,5]thiadiazole) was purchased from Derthon Optoelectronic Materials Science Technology Co LTD. All air and moisture sensitive reactions were carried out in flame-dried glassware under a nitrogen atmosphere.

Measurements. The UV-Vis-NIR absorption spectra were performed using a PerkinElmer Lambda 365 spectrophotometer. ^{1}H and ^{13}C spectra were recorded at room temperature on a Unity-400 NMR spectrometer using $CDCl_3$ as solvent and tetramethylsilane (TMS) as a reference. Mass spectra (MS) were measured with a GCT premier CAB048 mass spectrometer in MALDI-TOF mode. The photoluminescence (PL) spectra were conducted on a Horiba iHR 320 spectrofluorometer. Dynamic light scattering (DLS) was measured on a 90 plus particle size analyzer. Transmission electron microscopy (TEM) images were acquired from a JEM-2010F transmission electron microscope with an accelerating voltage of 200 kV. Density functional theory (DFT) calculations were carried out by the B3LYP/6G(d), Gaussian 09 package.

Synthetic procedures and characterization data for the compounds

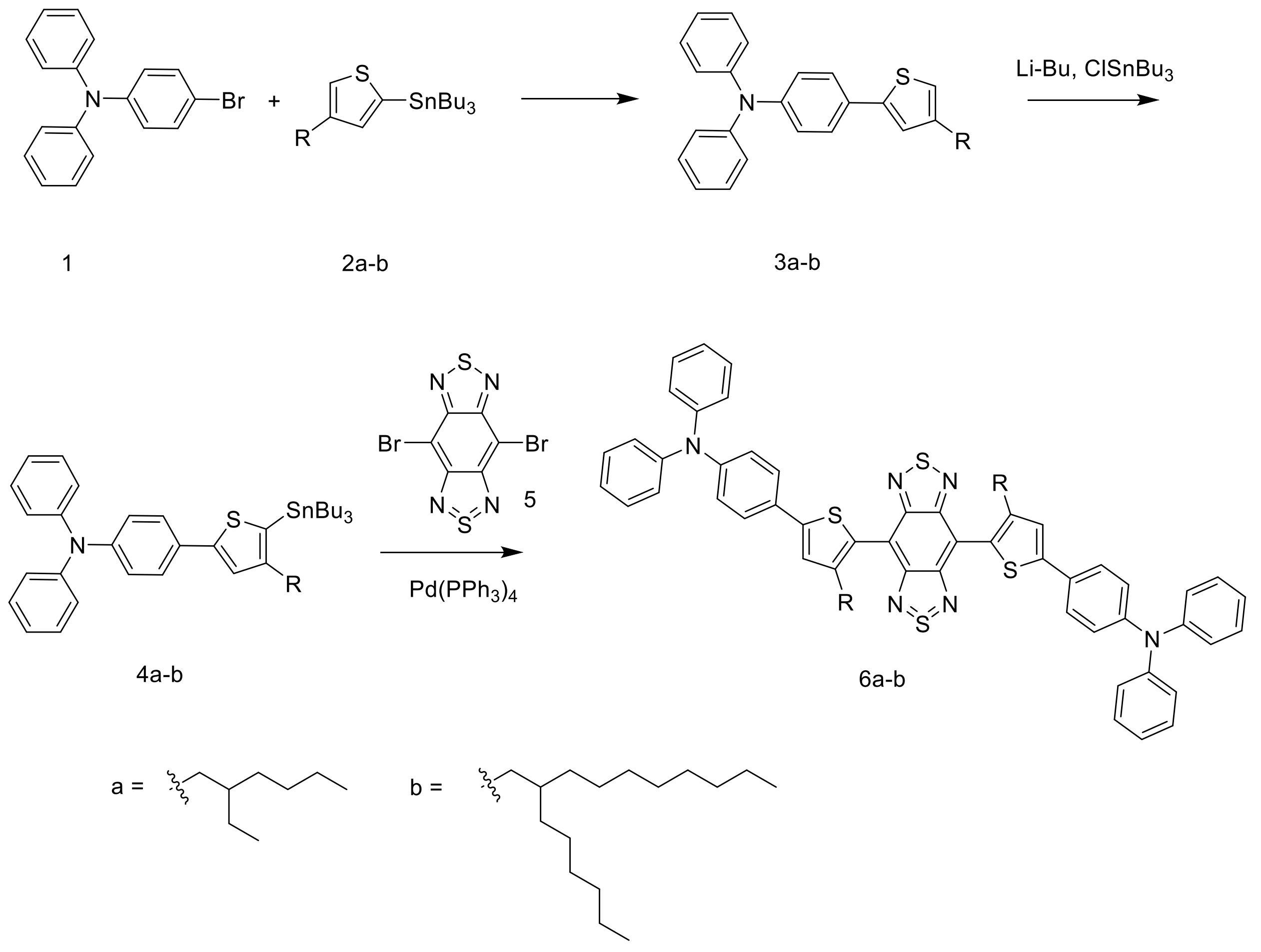

Supplementary Figure 1. Synthetic route to 2TT-*o*C26B (**6a**) and 2TT-*o*C610B (**6b**).

Synthetic route to 2TT-*o*C6B.

2TT-*o*C6B was synthesized according to the previous report.[1]

Synthetic route to 2a and 2b.

2a and **2b** were synthesized according to the previous report.[2]

Synthetic route to 3a.

Under N_2 atmosphere, **1** (1 g, 3.1 mmol), **2a** (1.5 g, 3.1 mmol), $Pd(PPh_3)_4$ (180 mg, 0.15 mmol), and 20 mL toluene were added to a 100 mL predried two-necked flask. The mixture was refluxed for 24 h. After cooling down to room temperature, the solvent was removed by rotary evaporation. The crude product was purified by silica gel column to obtain the target molecule (yield, 75%). ^1H NMR (400 MHz, $CDCl_3$) δ 7.48-7.45 (2H, m), 7.29-7.25 (3H, m), 7.13-7.01 (10H, m), 6.79 (1H, s), 2.55-2.53 (2H, d, J = 8 Hz), 1.64 (1H, m), 1.38-1.29 (8H, m), 0.89 (6H, m).

Synthetic route to 3b.

The synthetic route of **3b** was similar to **3a**, excepted that **2b** (1.85 g, 3.1 mmol) was used. ^1H NMR (400 MHz, $CDCl_3$) δ 7.48-7.46 (2H, m), 7.28-7.23 (3H, m), 7.13-7.01 (10H, m), 6.79 (1H, s), 2.55-2.53 (2H, d, J = 8 Hz), 1.65 (1H, m), 1.38-1.29 (24H, m), 0.89 (6H, m).

Synthetic route to 4a and 4b.

4a and **4b** were synthesized according to the previous report.[3]

Synthetic route to 2TT-*o*C26B (6a)

To a solution of compounds **5** (50 mg, 0.142 mmol) and **4a** (415 mg, 0.57 mmol) in toluene (10 mL) was added $Pd(PPh_3)_4$ (16 mg). The mixture was stirred for 48 h at 100 °C. After cooling down to room temperature, the mixture was poured into water and extracted with DCM. The organic layer was washed with saturated KF and brine before being dried over $MgSO_4$. After evaporation of the solvent, the residue was purified by column chromatography on silica gel to afford product (yield: 30%). ^1H NMR (400 MHz, $CDCl_3$), δ (ppm) = 7.59-7.57 (4H, m), 7.31-7.29 (10H, m), 7.16-7.04 (16H, m), 2.59-2.57 (4H, d, J = 8Hz), 1.35 (2H, m), 1.15-1.10 (16H, m), 0.73 (12H, m). ^{13}C NMR (100 MHz, $CDCl_3$), δ (ppm): 152.53, 146.82, 145.99, 144.02, 128.70, 128.12, 127.55, 126.12, 124.00, 122.78, 122.55, 115.77, 39.96, 33.81, 31.84, 27.96, 24.97, 22.15, 13.38, 9.99. MS: m/z: [M]+ calcd for $C_{66}H_{64}N_6S_4$: 1068.4075, found: 1068.4070.

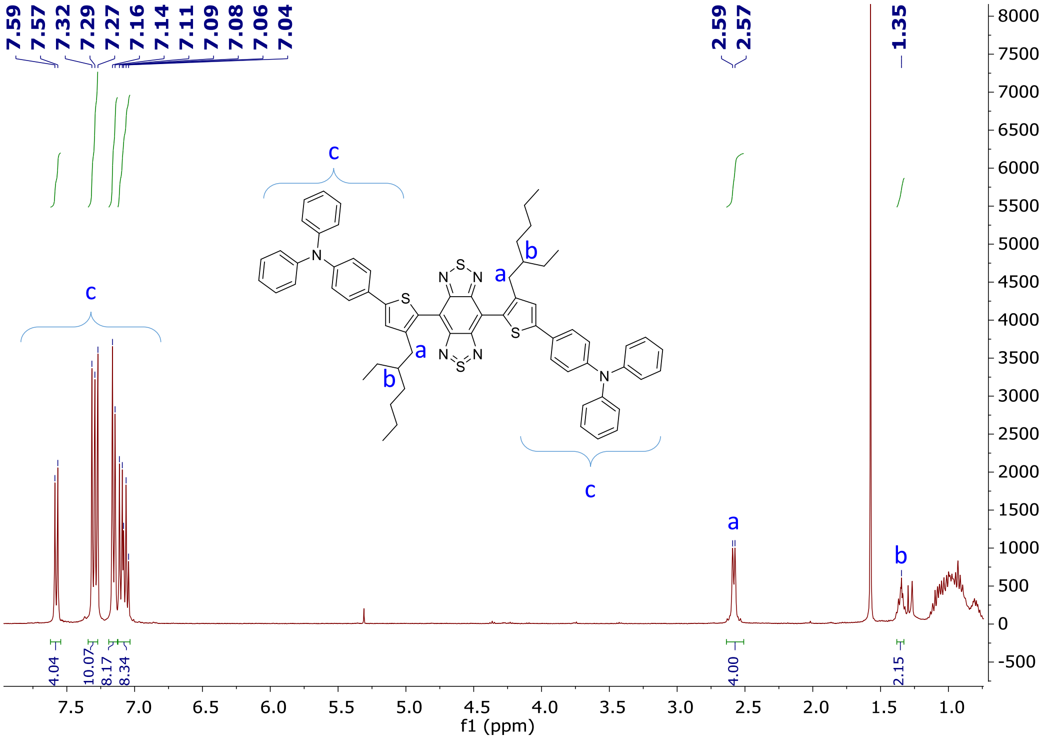

Supplementary Figure 2. ^{1}H NMR spectrum of **2TT-*o*C26B (6a).**

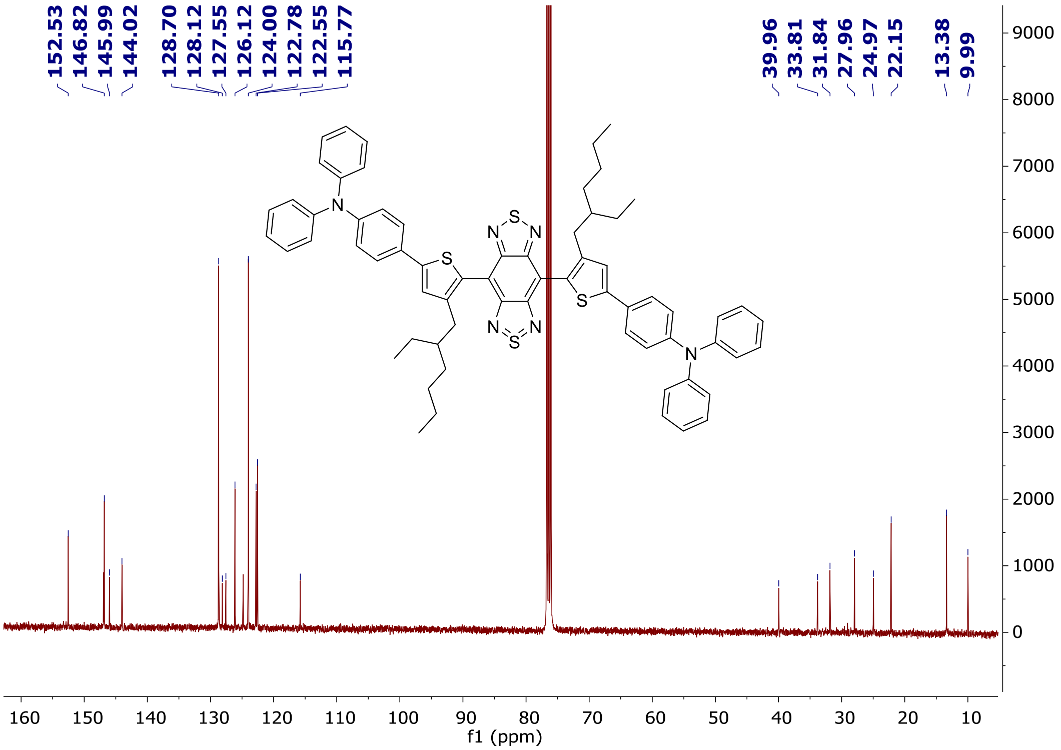

Supplementary Figure 3. ^{13}C NMR spectrum of **2TT-*o*C26B (6a).**

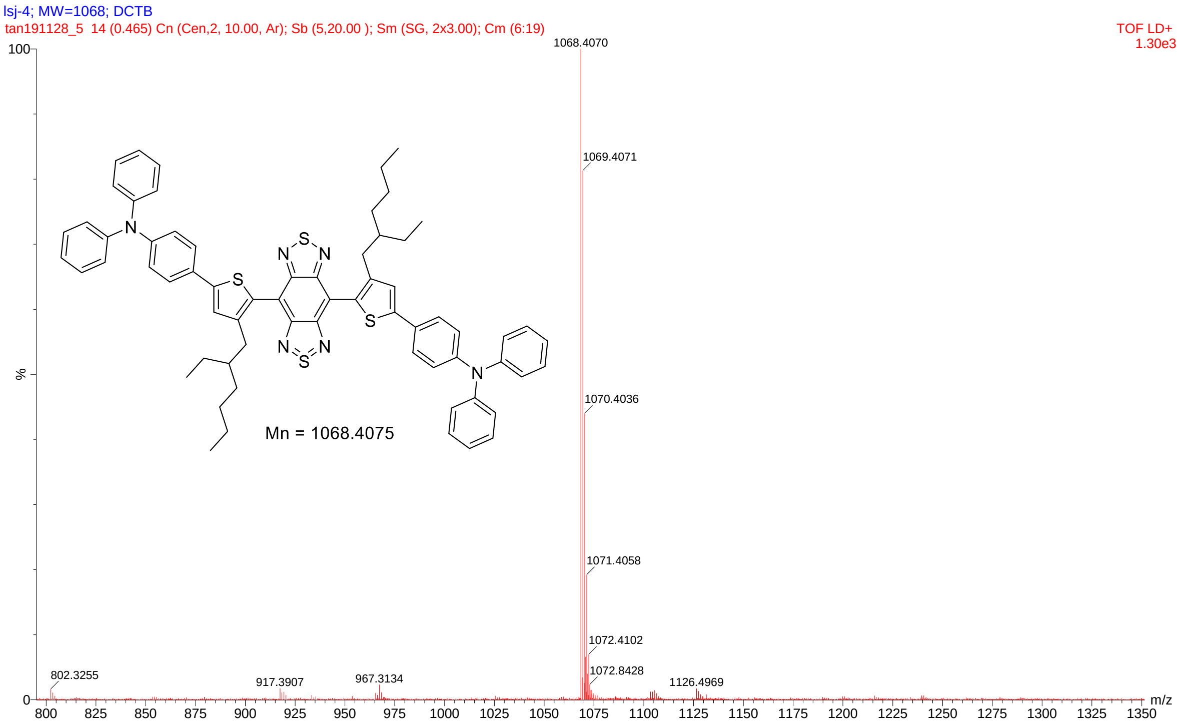

Supplementary Figure 4. MALDI-TOF-MS spectrum of **2TT-*o*C26B (6a).**

Synthetic route to 2TT-*o*C610B (6b)

The synthetic route of **2TT-*o*C610B (6b)** was similar to **6a**. ^{1}H NMR (400 MHz, $CDCl_3$), δ (ppm) = 7.59-7.56 (4H, m), 7.31-7.27 (10H, m), 7.16-7.04 (16H, m), 2.58-2.57 (4H, d, J = 4 Hz), 1.44-1.43 (2H, m), 1.27-0.93 (48H, m), 0.83 (12H, m). ^{13}C NMR (100 MHz, $CDCl_3$), δ (ppm): 152.53, 146.92, 145.96, 144.01, 128.69, 128.16, 127.59, 126.08, 124.77, 123.98, 122.79, 122.53, 115.77, 38.61, 34.17, 32.61, 31.26, 29.23, 28.93, 25.78, 25.72, 22.05, 13.49. MS: m/z: [M]+ calcd for $C_{82}H_{96}N_6S_4$: 1292.6579, found: 1292.6530.

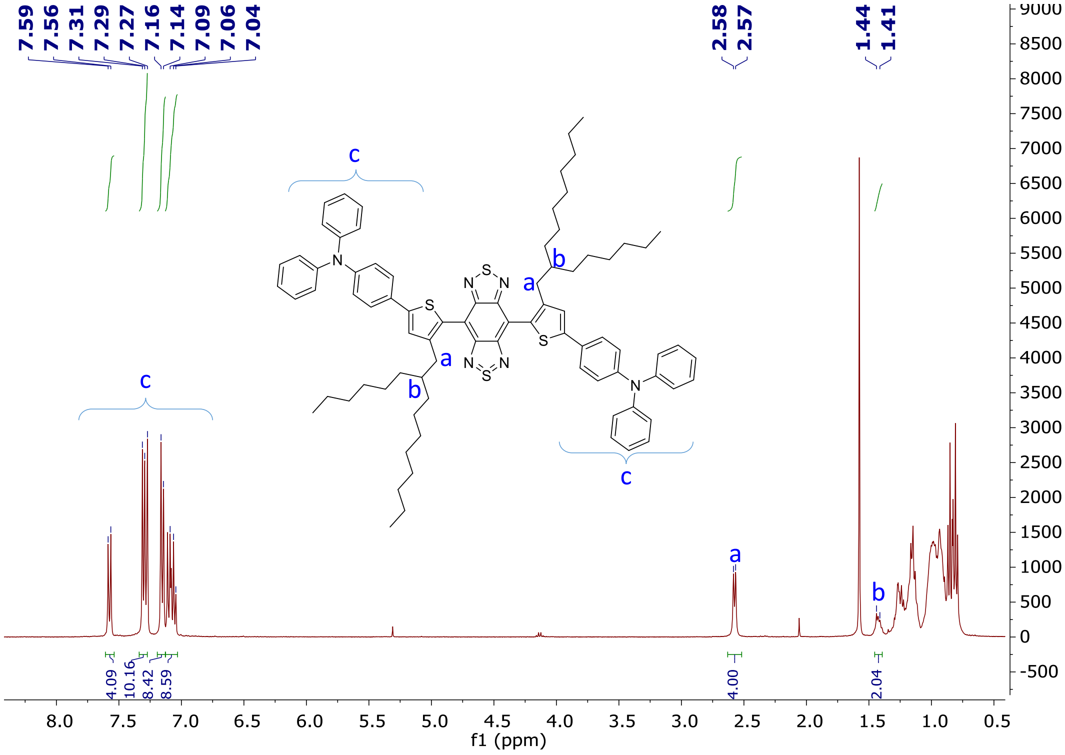

Supplementary Figure 5. ^{1}H NMR spectrum of **2TT-*o*C610B (6b)**.

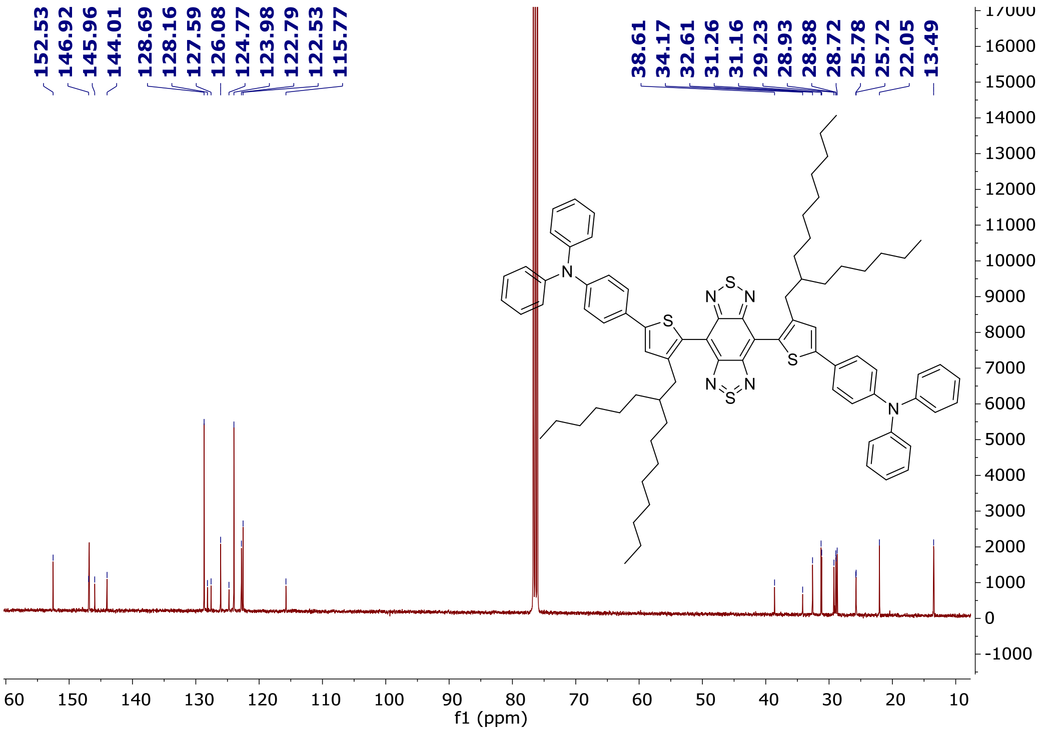

Supplementary Figure 6. ^{13}C NMR spectrum of **2TT-*o*C610B (6b)**.

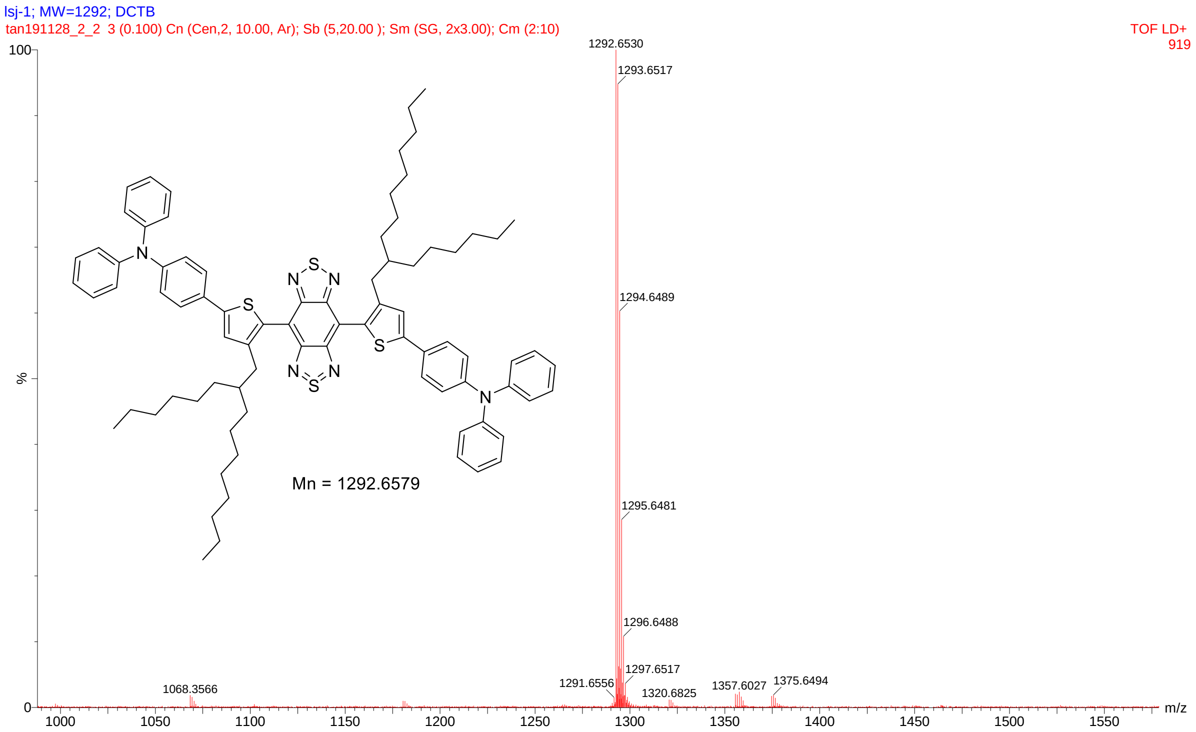

Supplementary Figure 7. MALDI-TOF-MS spectrum of **2TT-*o*C610B (6b)**.

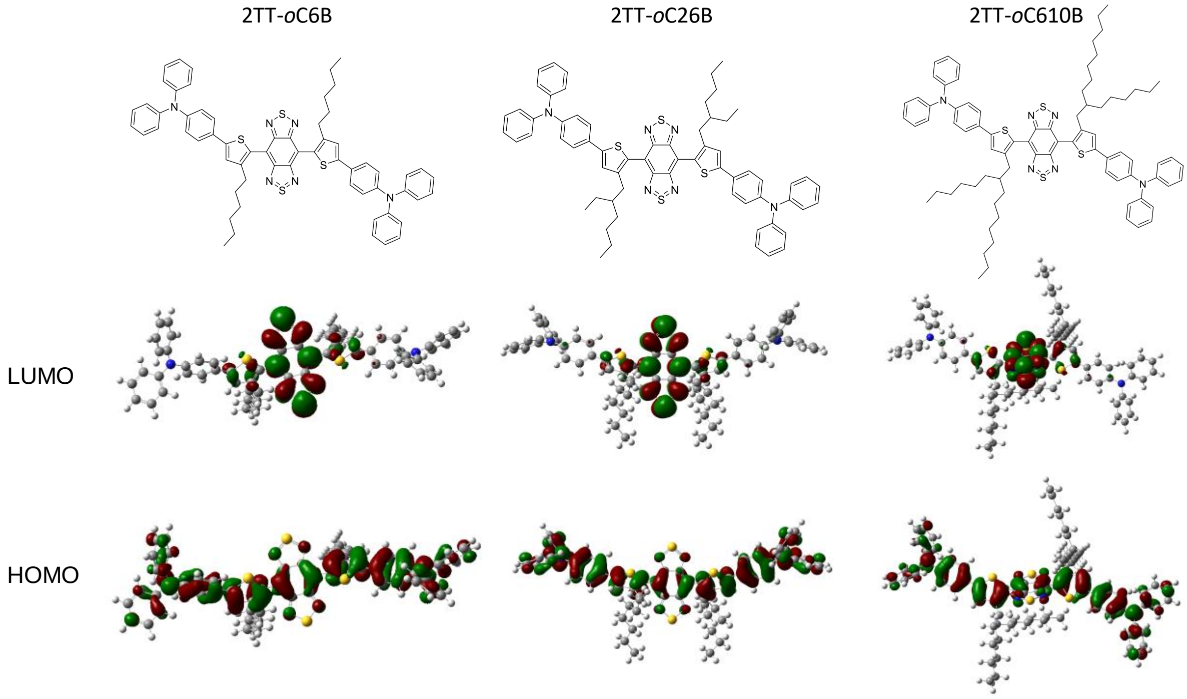

Supplementary Figure 8. Calculated HOMOs and LUMOs of the compounds.

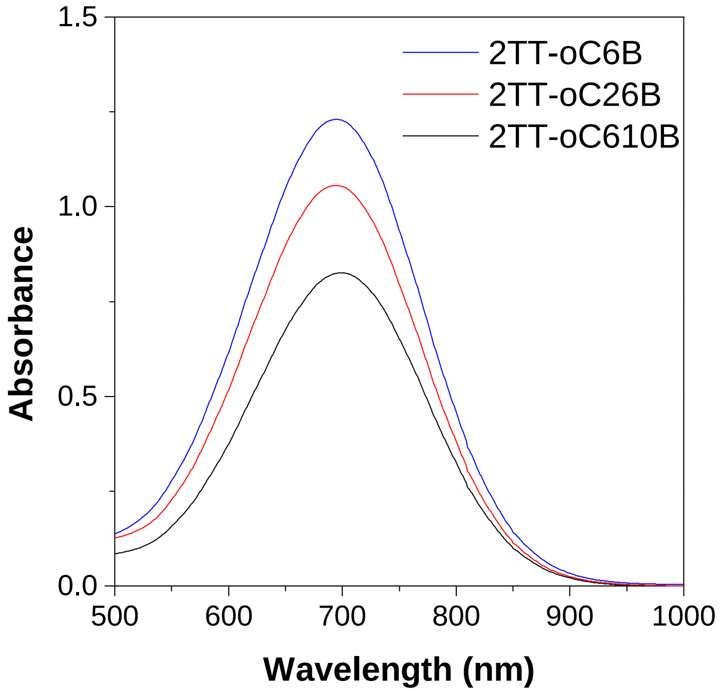

Supplementary Figure 9. Absorbance spectra of the molecules in THF (0.05 mg/mL). The molar absorption coefficient of 2TT-oC6B, 2TT-oC26B and 2TT-oC610B in THF is 2.49×10^4, 2.25×10^4, 2.13×10^4 L mol^{-1} cm^{-1}, respectively.

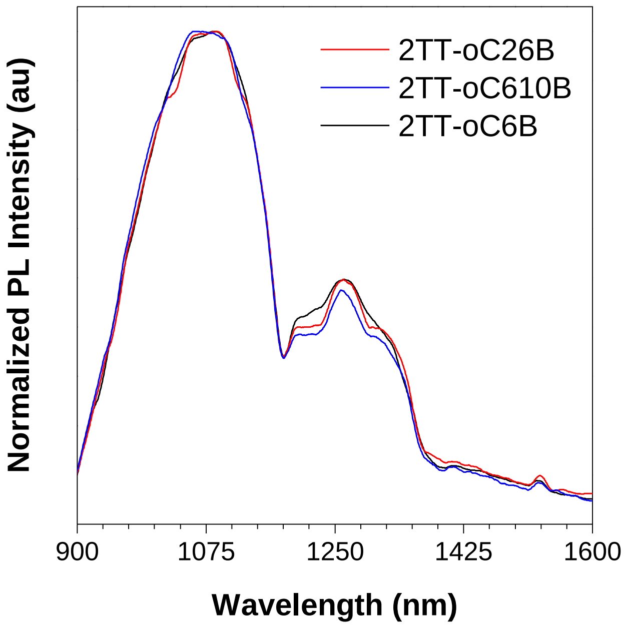

Supplementary Figure 10. Normalized PL spectra of the molecules in THF.

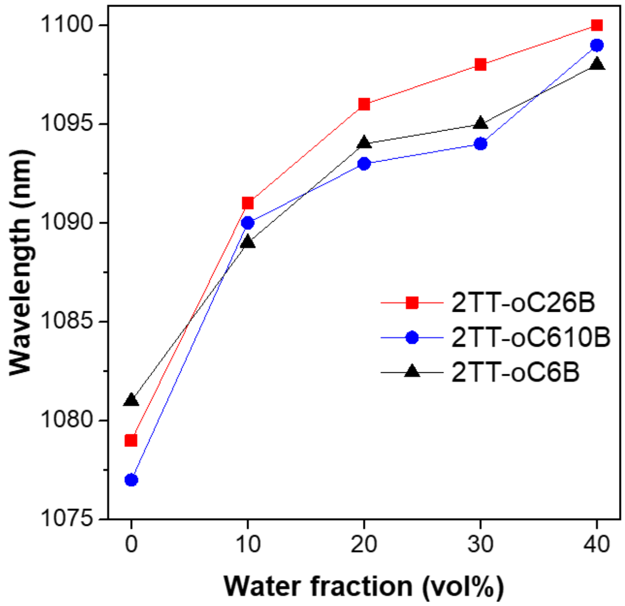

Supplementary Figure 11. The emission wavelength variation of the molecules with different water fraction from 0 to 40%.

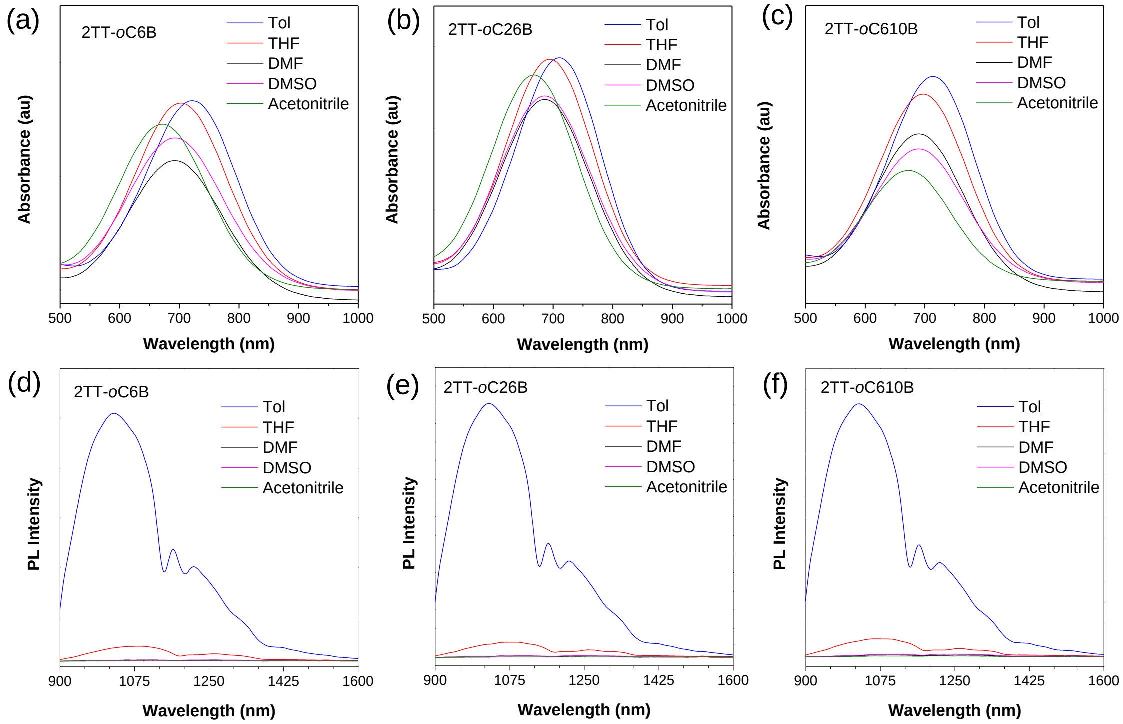

Supplementary Figure 12. The absorption and emission spectra of the molecules in different solvents at the same concentration.

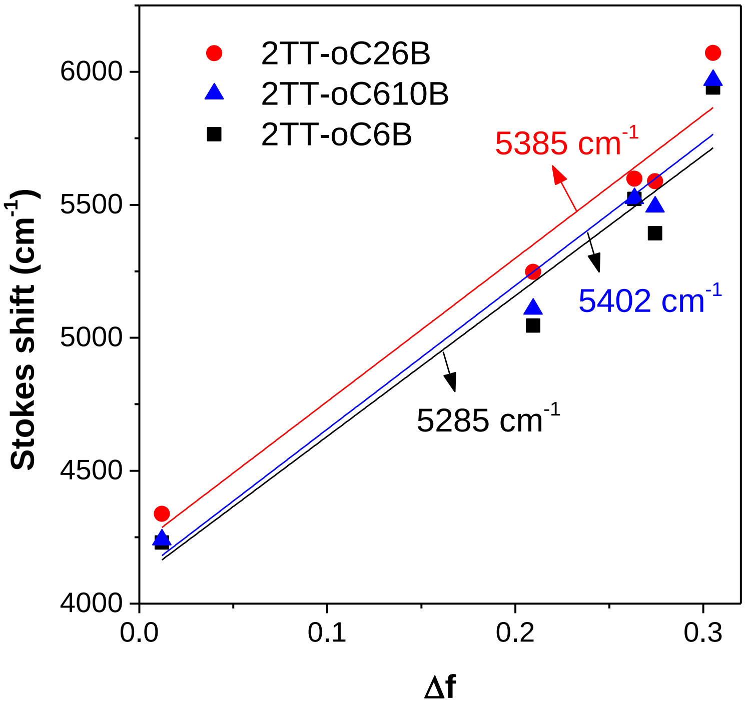

Supplementary Figure 13. Correlation of solvent polarity parameter with Stokes shift.

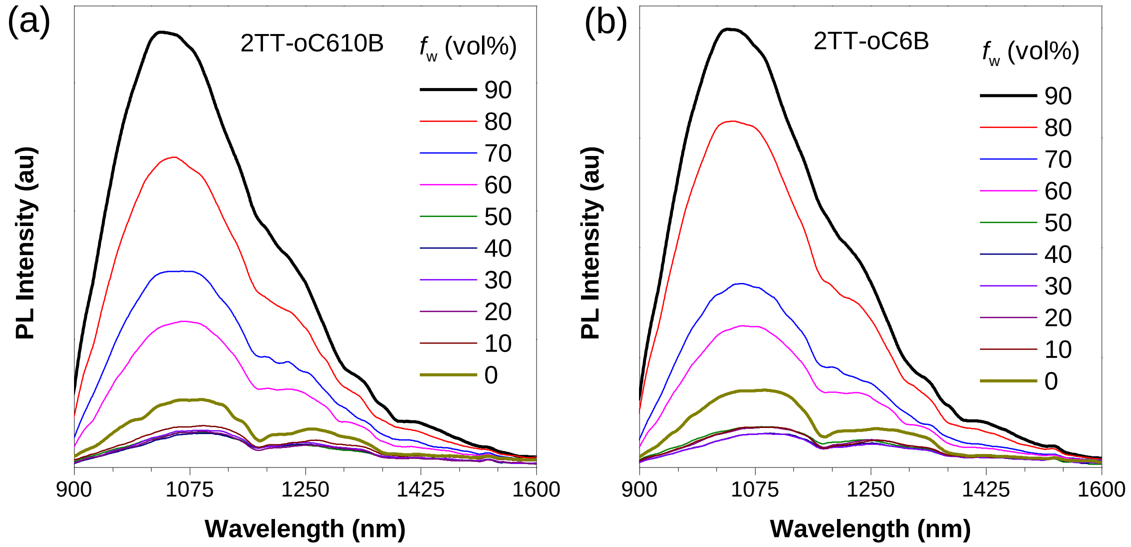

Supplementary Figure 14. Variation of PL intensity of 2TT-*o*C610B (a) and 2TT-*o*C6B (b) with water fraction in THF/water mixtures.

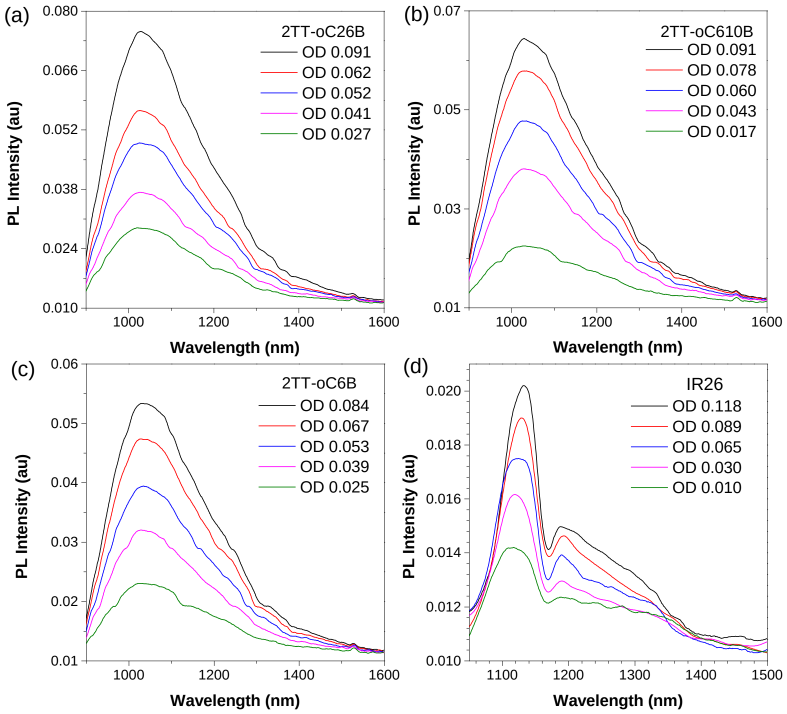

Supplementary Figure 15. NIR-II quantum yield measurement of nanoparticles. NIR-II fluorescence emission of nanoparticles (a, 2TT-*o*C26B; b, 2TT-*o*C610B; c, 2TT-oC6B) and (d) IR26 (DCE) with increasing concentrations. Source data are provided as a Source Data file.

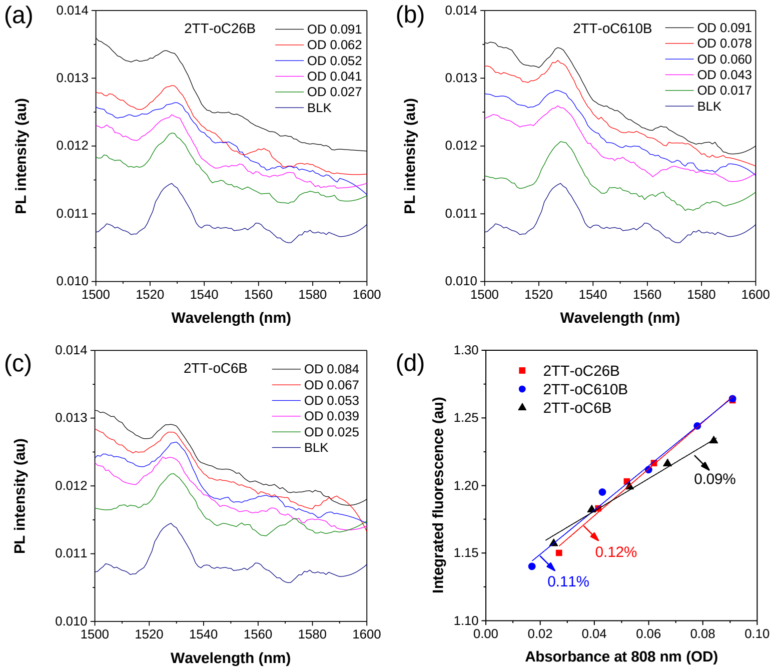

Supplementary Figure 16. NIR-IIb quantum yield measurement of nanoparticles. NIR-II fluorescence emission of nanoparticles in the NIR-IIb region (1500-1600 nm) with increasing concentrations. (a, 2TT-

*o*C26B; b, 2TT-*o*C610B; c, 2TT-*o*C6B). (d) The plots for the integrated fluorescence spectra of the three compounds nanoparticles in the NIR-IIb region (1500-1600 nm) at five different concentrations. BLK: pure water. Source data are provided as a Source Data file.

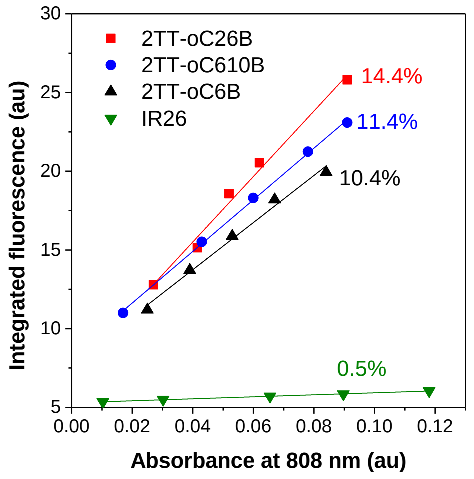

Supplementary Figure 17. The plots for the integrated fluorescence spectra of the three compounds nanoparticles (900-1600 nm) and IR26 (1050-1500 nm) (reference, QY = 0.5% in dichloroethane) at five different concentrations. Source data are provided as a Source Data file.

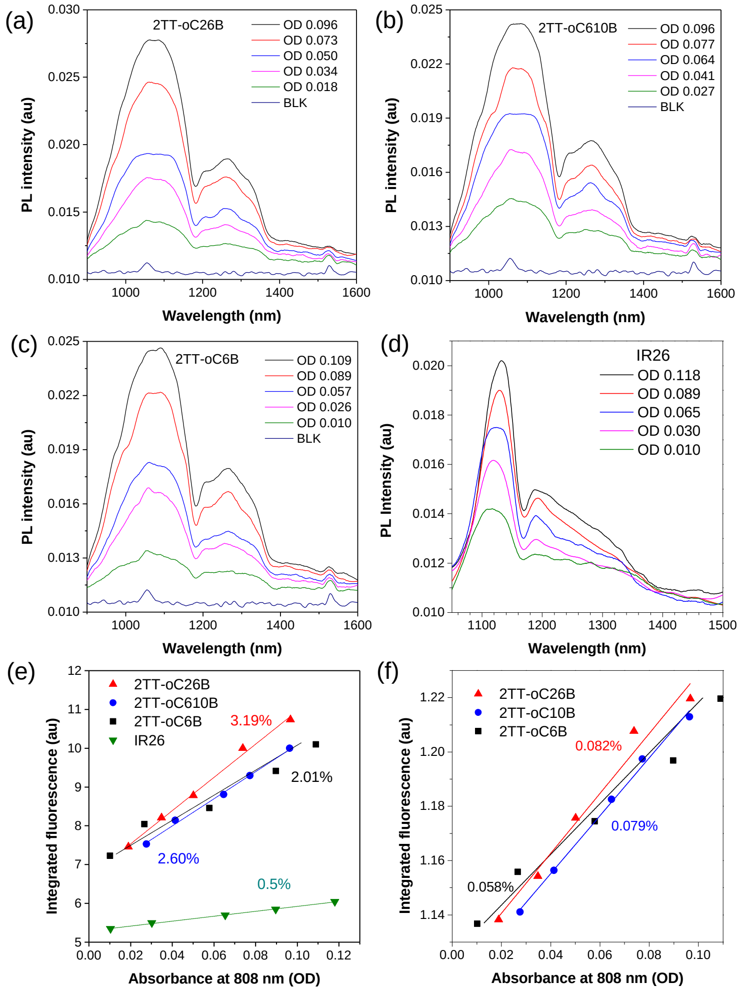

Supplementary Figure 18. Quantum yield measurement in THF. NIR-II fluorescence emission of molecules in solvent (a, 2TT-*o*C26B; b, 2TT-*o*C610B; c, 2TT-*o*C6B in THF) and (d) IR26 (in dichloroethane) with increasing concentrations. BLK: pure THF. (e) The plots for the integrated fluorescence spectra of the three compounds (in THF) and IR26 (reference, QY = 0.5% in dichloroethane) at five different concentrations. The NIR-II (1000-1600 nm) QY of 2TT-*o*C26B, 2TT-*o*C610B and 2TT-*o*C6B was 3.19%, 2.60% and 2.01%, respectively. (f) The plots for the integrated fluorescence spectra of the three compounds in the NIR-IIb (1500-1600 nm) region at five different concentrations. The NIR-IIb QY of 2TT-*o*C26B, 2TT-*o*C610B and 2TT-*o*C6B was 0.082%, 0.079% and 0.058%, respectively.

Supplementary Table 1. The summary of QYs measured in water and THF.

Molecules	Nanoparticles (900-1600 nm)		Nanoparticles (1000-1600 nm)		Nanoparticles (1500-1600 nm)		THF (1000-1600 nm)		THF (1500-1600 nm)	
	Slope	QY (%)	Slope	QY (%)	Slope	QY (%)	Slope	QY (%)	Slope	QY (%)
2TT-oC26B	207	14.4	164.38	11.5	1.72	0.120	42.8	3.19	1.103	0.082
2TT-oC610B	164	11.4	130.57	9.1	1.63	0.114	34.9	2.60	1.060	0.079
2TT-oC6B	149	10.4	120.89	8.4	1.26	0.088	26.9	2.01	0.784	0.058

Note: The slope of IR-26 is 6.35 according to Fig. 3d, Supplementary Fig. 15d and 18d.

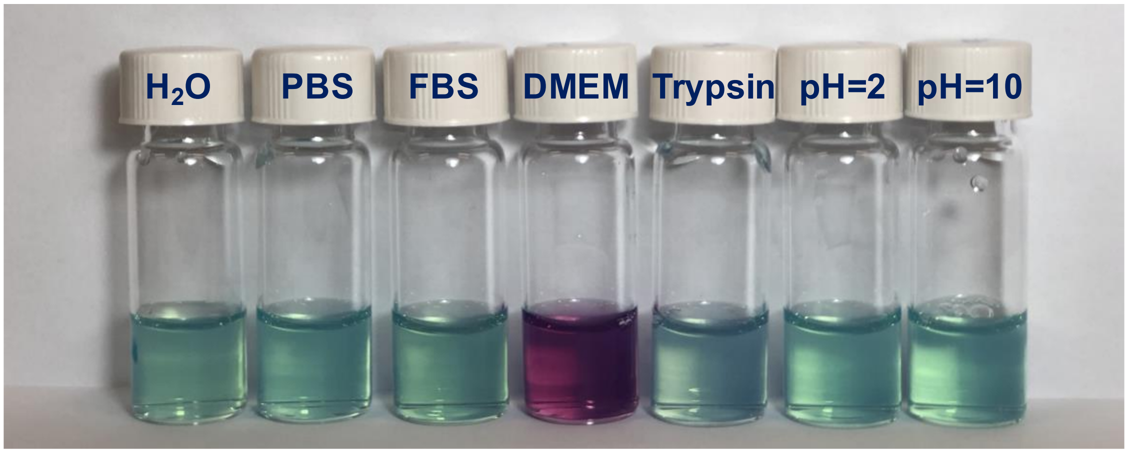

Supplementary Figure 19. The photo of 2TT-*o*C26B NPs in different solution after 5 days.

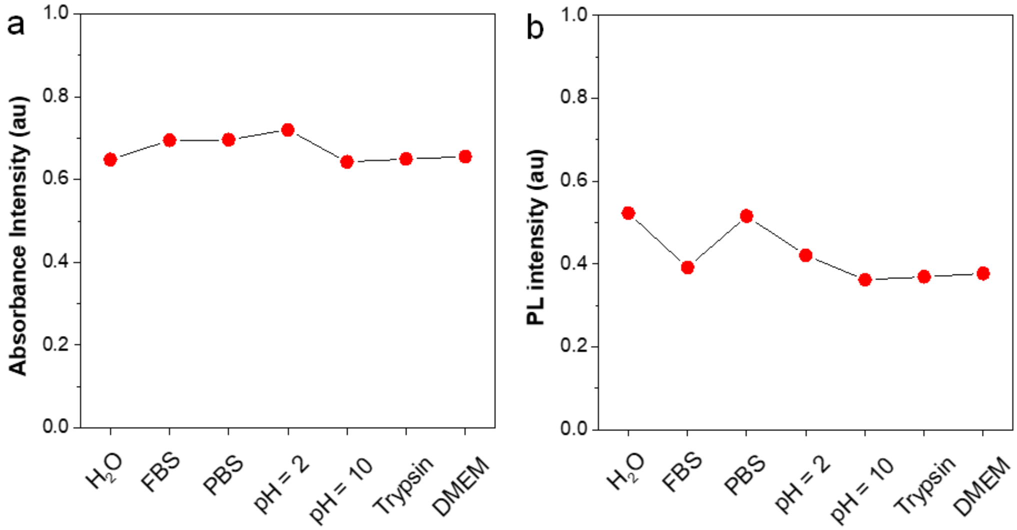

Supplementary Figure 20. The (a) absorption and (b) emission change of 2TT-*o*C26B NPs in different solution after 5 days.

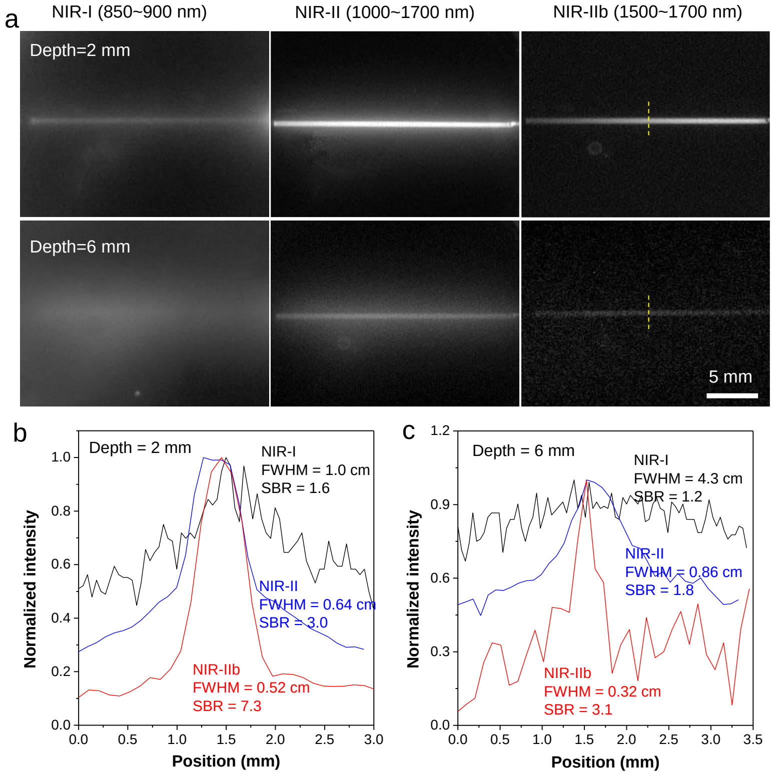

Supplementary Figure 21. (a) NIR fluorescence images of a capillary tube filled with 2TT-*o*C26B NPs immersed at depths of 2 mm (top) and 6 mm (bottom) in 1% Intralipid, recorded in NIR-I, NIR-II and NIR-IIb regions, respectively. (b, c) Cross-sectional fluorescence intensity profiles along yellow-dashed lines in the middle of capillary tubes. b: depth=2 mm, c: depth=6 mm.

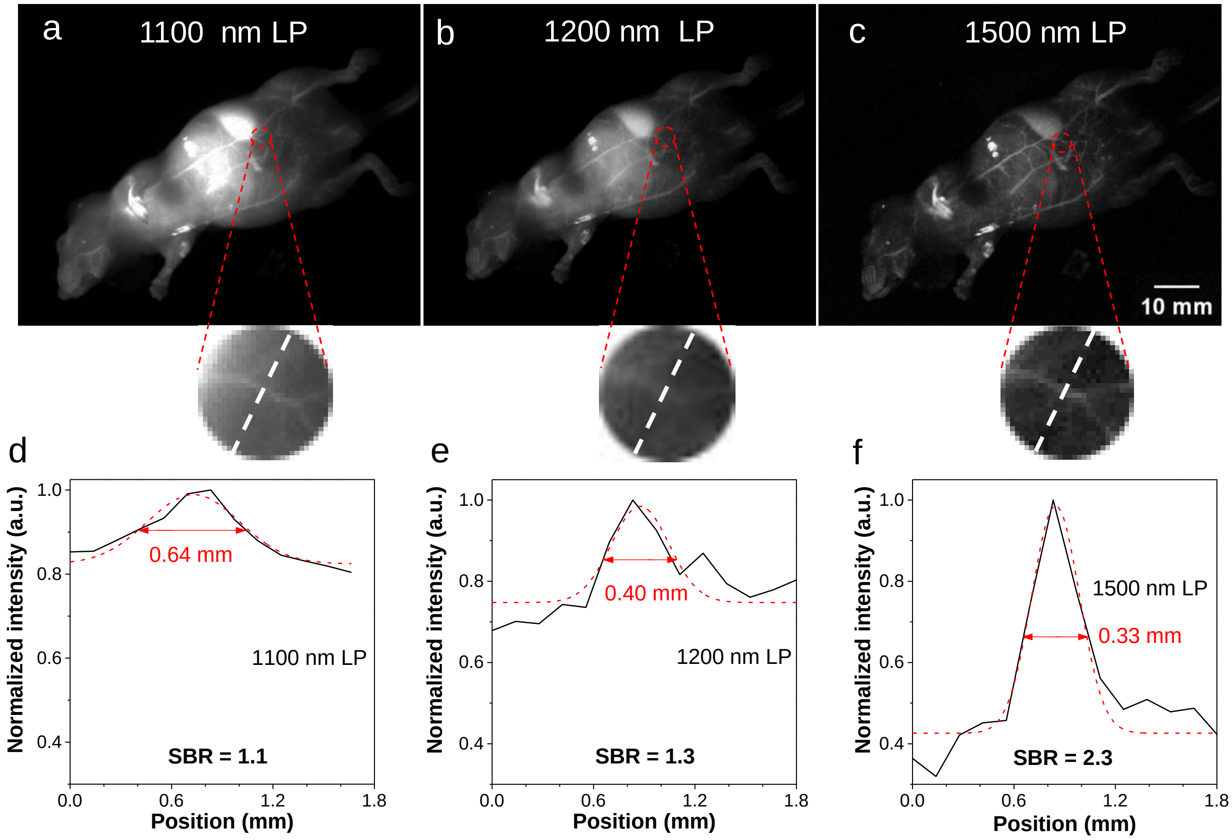

Supplementary Figure 22. Comparison of NIR-II fluorescence signals for whole-body imaging of living mice under different LP filters treated with 2TT-*o*C26B NPs (793 nm excitation). (a) 1100 nm LP, 5ms, 37 mW/cm^2; (b) 1200 nm LP, 5 ms, 37 mW/cm^2; (c) 1500 nm LP, 150 ms, 75 mW/cm^2. (d-f) Corresponding cross-sectional fluorescence intensity profiles along white-dashed lines. Gaussian fits to the profile are shown in the red line.

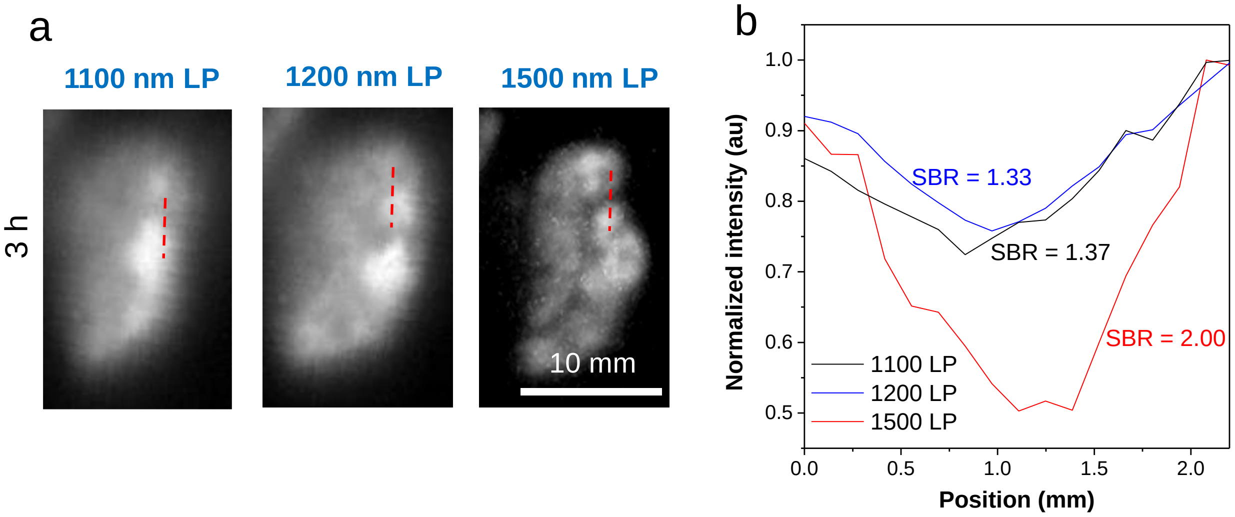

Supplementary Figure 23. (a) Zoom-in images of the yellow rectangle area marked in Fig. 7a (3 h). (b) A cross-sectional fluorescence intensity profile along the red-dashed bar in the top of (b).

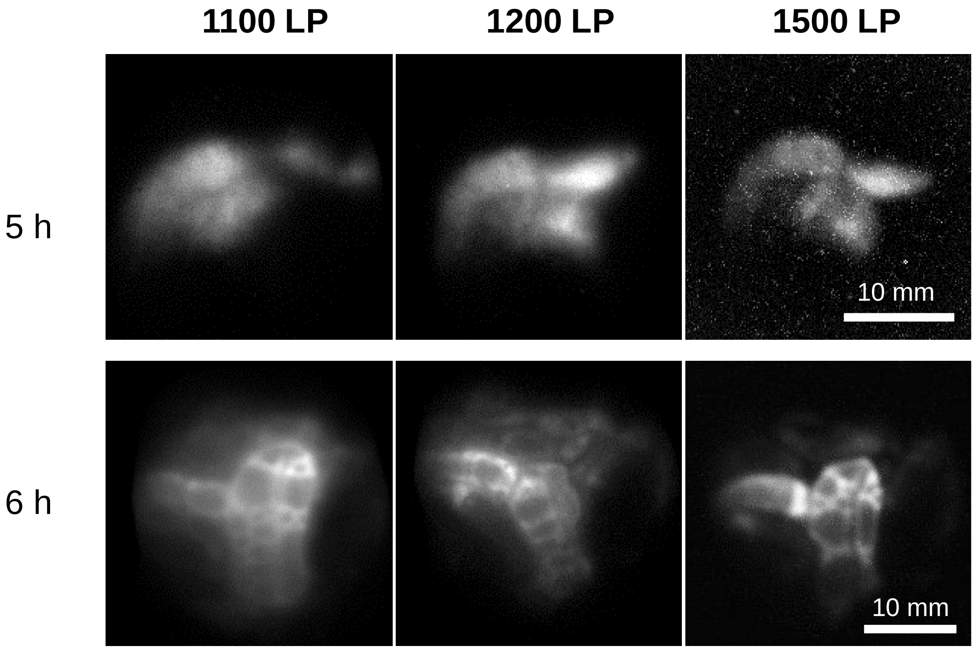

Supplementary Figure 24. The zoom-in images of the two yellow rectangle areas marked in Fig. 7a (5 and 6 h).

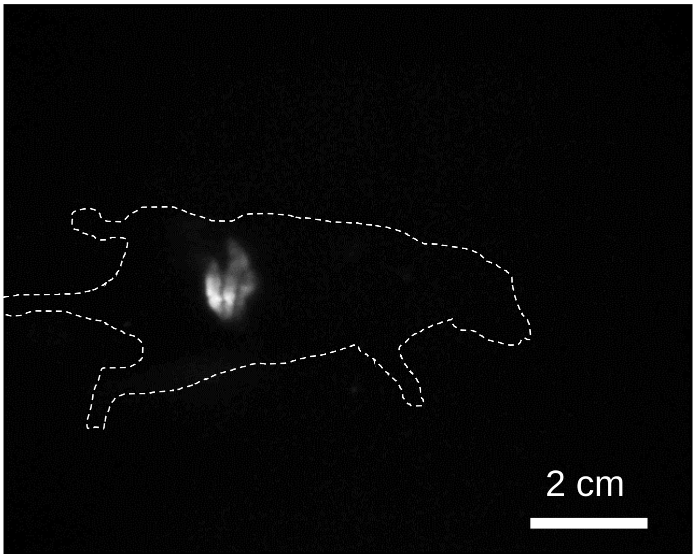

Supplementary Figure 25. Real-time imaging of intestinal peristalsis in living mice gavaged with the 2TT-*o*C26B NPs (300 μL, 1 mg/mL) at NIR-IIb region (793 nm laser excitation 250 mW/cm^2 within safety limits[4], exposure time: 40 ms).

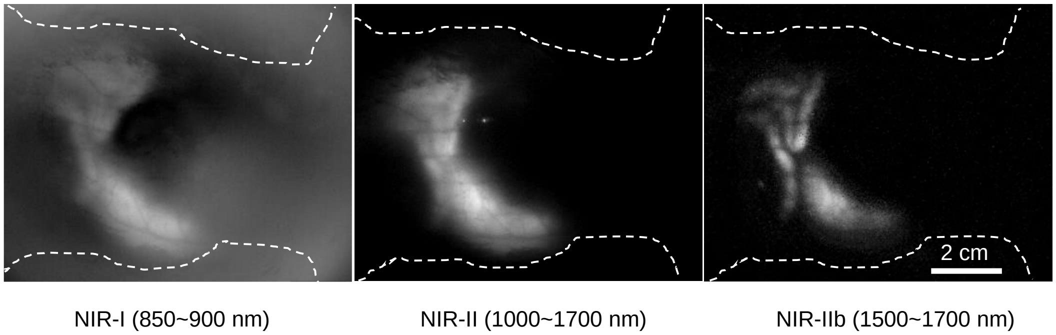

Supplementary Figure 26. NIR fluorescence images of intestinal tract in living rat recorded in NIR-I, NIR-II and NIR-IIb regions. Rat was imaged at 5 hours after being gavage with the 2TT-*o*C26B NPs (1 mg/mL, 3 mL) under a 793 nm laser excitation with a power intensity less than 250 mW/cm^2.

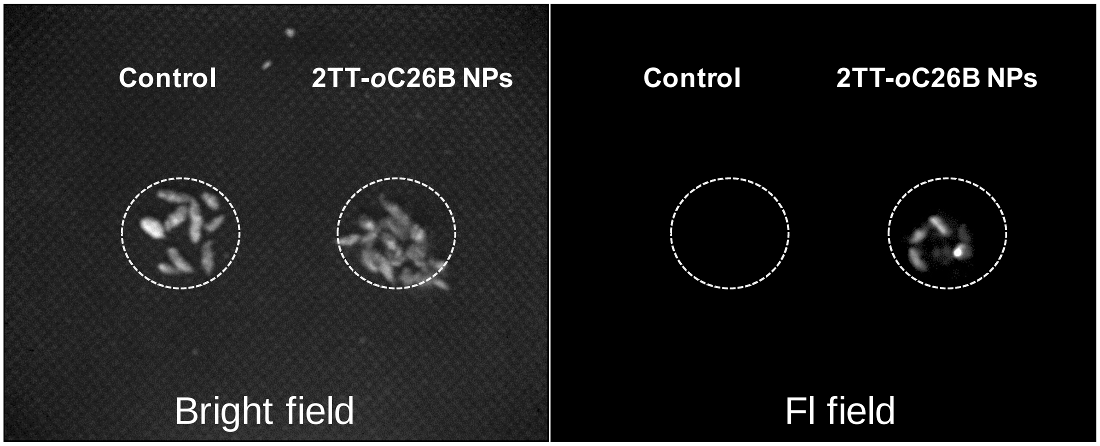

Supplementary Figure 27. Feces images of mice under bright field (left) and fluorescence field (right). Feces from mice without 2TT-*o*C26B NPs were used as control.

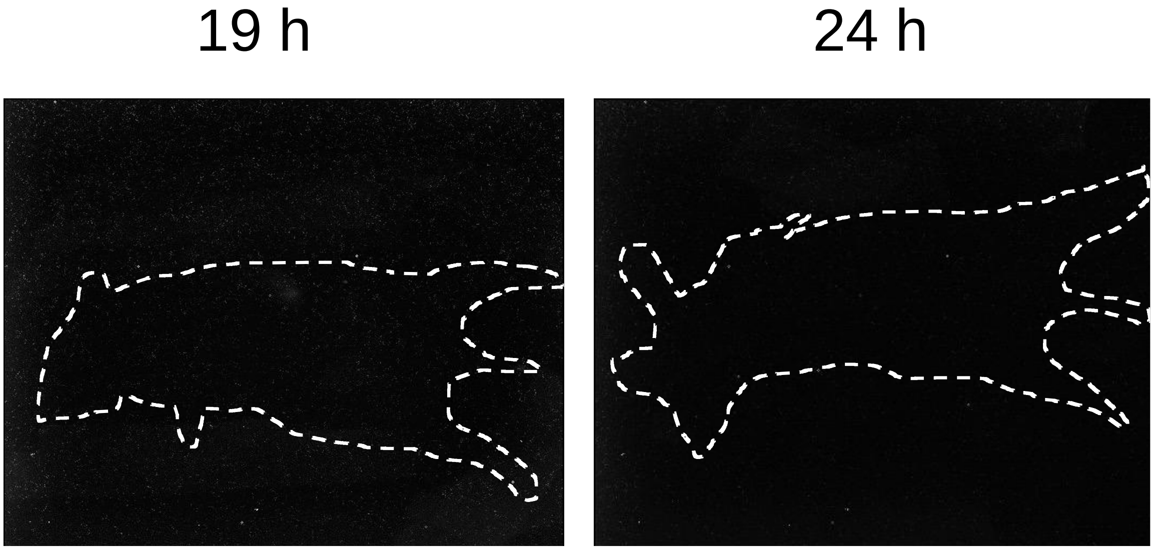

Supplementary Figure 28. Real-time monitoring of intestinal peristalsis in living mice gavaged with the 2TT-*o*C26B NPs (300 μL, 1 mg/mL) using 1500 nm LP filter (75 ms, 110 mW/cm^2) at 19 and 24 h, respectively. The disappearance of NIR-II signal indicated that 2TT-*o*C26B NPs were entirely excreted from the body, which is crucial for biosafety.

Supplementary References

1. Liu, S. et al. Constitutional isomerization enables bright NIR-II AIEgen for brain-inflammation imaging. *Adv. Funct. Mater.*, 1908125 (2019).
2. Liu, Y. et al. Aggregation and morphology control enables multiple cases of high-efficiency polymer solar cells. *Nat. Commun.* **5**, 5293 (2014).
3. Liu, S. et al. Molecular motion in aggregates: manipulating TICT for boosting photothermal theranostics. *J. Am. Chem. Soc.* **141**, 5359-5368 (2019).
4. Lin, J. et al. Novel near-infrared II aggregation-induced emission dots for in vivo bioimaging. *Chem. Sci.* **10**, 1219-1226 (2019).
