# Supplementary material for: Design of AIEgens for near-infrared IIb imaging through structural modulation at molecular and morphological levels
Source: Nat Commun. 2020 Mar 9;11:1255. doi: 10.1038/s41467-020-15095-1 (PMC7062876; doi:10.1038/s41467-020-15095-1)
Supplement: Supplementary file 3 — Description of Additional Supplementary Files [file 41467_2020_15095_MOESM3_ESM.pdf]

Description of Additional Supplementary Files

Title: Supplementary Movie 1.

Description: NIRIIb fluorescence imaging of the contractile function of the intestine
